# Immunity following SARS-CoV-2 vaccination in autoimmune neurological disorders treated with rituximab or ocrelizumab

**DOI:** 10.3389/fimmu.2023.1149629

**Published:** 2023-06-16

**Authors:** Petra Nytrova, Dominika Stastna, Adam Tesar, Ingrid Menkyova, Helena Posova, Helena Koprivova, Veronika Mikulova, Jiri Hrdy, Gabriela Smela, Dana Horakova, Irena Rysankova, Kristyna Doleckova, Michaela Tyblova

**Affiliations:** ^1^ Department of Neurology and Centre of Clinical Neuroscience, First Faculty of Medicine, Charles University and General University Hospital in Prague, Prague, Czechia; ^2^ Institute of Biophysics and Informatics of the First Faculty of Medicine, Charles University in Prague, Prague, Czechia; ^3^ 2nd Department of Neurology, Faculty of Medicine, Comenius University, Bratislava, Slovakia; ^4^ Laboratory of Clinical Immunology and Allergology, Institute of Clinical Biochemistry and Laboratory Diagnostics, First Faculty of Medicine, Charles University and General University Hospital in Prague, Prague, Czechia; ^5^ Institute of Immunology and Microbiology, First Faculty of Medicine, Charles University and General University Hospital in Prague, Prague, Czechia

**Keywords:** SARS-CoV-2 mRNA vaccine, SARS-CoV-2, humoral immune response, IFN gamma release assay, myasthenia gravis, multiple sclerosis, neuromyelitis optica spectrum disorder

## Abstract

**Background:**

Rituximab (RTX) and ocrelizumab (OCR), B cell-depleting therapy targeting CD20 molecules, affect the humoral immune response after vaccination. How these therapies influence T-cell-mediated immune response against SARS-CoV-2 after immunization remains unclear. We aimed to evaluate the humoral and cellular immune response to the COVID-19 vaccine in a cohort of patients with multiple sclerosis (MS), neuromyelitis optica spectrum disorders (NMOSD), and myasthenia gravis (MG).

**Methods:**

Patients with MS (83), NMOSD (19), or MG (7) undergoing RTX (n=47) or OCR (n=62) treatment were vaccinated twice with the mRNA BNT162b2 vaccine. Antibodies were quantified using the SARS-CoV-2 IgG chemiluminescence immunoassay, targeting the spike protein. SARS-CoV-2-specific T cell responses were quantified by interferon γ release assays (IGRA). The responses were evaluated at two different time points (4-8 weeks and 16-20 weeks following the 2nd dose of the vaccine). Immunocompetent vaccinated individuals (n=41) were included as controls.

**Results:**

Almost all immunocompetent controls developed antibodies against the SARS-CoV-2 trimeric spike protein, but only 34.09% of the patients, without a COVID-19 history and undergoing anti-CD20 treatment (via RTX or OCR), seroconverted. This antibody response was higher in patients with intervals of longer than 3 weeks between vaccinations. The duration of therapy was significantly shorter in seroconverted patients (median 24 months), than in the non-seroconverted group. There was no correlation between circulating B cells and the levels of antibodies. Even patients with a low proportion of circulating CD19^+^ B cells (<1%, 71 patients) had detectable SARS-CoV-2 specific antibody responses. SARS-CoV-2 specific T cell response measured by released interferon γ was detected in 94.39% of the patients, independently of a humoral immune response.

**Conclusion:**

The majority of MS, MG, and NMOSD patients developed a SARS-CoV-2-specific T cell response. The data suggest that vaccination can induce SARS-CoV-2-specific antibodies in a portion of anti-CD20 treated patients. The seroconversion rate was higher in OCR-treated patients compared to those on RTX. The response represented by levels of antibodies was better in individuals, with intervals of longer than 3 weeks between vaccinations.

## 1 Introduction

Anti-CD20-based B-cell-depleting strategies are implemented in treatment schemes for different autoimmune neurological disorders including multiple sclerosis (MS), neuromyelitis optica spectrum disorders (NMOSD), and myasthenia gravis (MG) (1, 2). Unfortunately, treatment with rituximab and ocrelizumab (monoclonal anti-CD20 antibodies) has been shown to be one of the risk factors for a severe course of COVID-19 infection in MS, NMOSD, and MG patients (3–5). Considering these findings, the vaccination against the SARS-CoV-2 virus promised reduction in the severity of COVID-19 in anti-CD20 treated patients.

Several questions have been posed, regarding the effect on humoral and cellular responses to vaccination based on the effect of rituximab (RTX) and ocrelizumab (OCR) on CD20^pos^ B-cells and T cells. A limited humoral response upon vaccination has already been demonstrated in several studies focusing on neurological patients (6–10). On the other hand, several studies have also shown evidence for the preserved cellular response of MS patients treated with RTX or OCR to vaccination against SARS-CoV-2, but there is a paucity of information about the longevity of these responses (11–14). Data on the cellular immune response induced by vaccination in patients with NMOSD and MG on anti-CD20 therapy are limited. Moreover, we still have some uncertainty in the interpretation of laboratory results and identification of parameters of immune responses predicting sufficient and effective protection against the severe course of COVID-19.

Neutralizing antibodies play a role in protection against SARS-CoV-2 and an orchestrated adaptive immunity can limit COVID19 severity. This implies, that the B cell immune response deficit in the patients treated with anti-CD20 drugs, results in impaired clearance of SARS-CoV-2, hence, a higher risk of severe and/or prolonged symptomatology (10, 15). The effective T cell immune response was shown to be associated with a milder COVID-19 course (15, 16). The immune response to vaccination can be affected by different factors, including different immunosuppressive treatments, age, and gender (17). B cell activation is a key factor contributing to the efficacy of the vaccine response, but there are several age-related changes in B cells, such as decreased plasma cells and IgM memory cells, which may contribute to the loss of vaccine efficacy (18). This factor should be considered in evaluating the vaccination effectiveness in older patients treated with immunosuppressive drugs or their combination in the context of a neurological diagnosis.

In this study, we analysed a cohort of patients with MS, NMOSD and MG treated with anti-CD20 therapy, compared with healthy controls, to evaluate the impact of rituximab or ocrelizumab therapy on the efficacy of the SARS-CoV-2 mRNA vaccine in eliciting humoral and cellular immunity, measured by detection of specific antibodies against the spike protein and release of interferon-gamma (IFN-γ) after spike protein stimulation of blood cells, respectively. Furthermore, we tried to evaluate other parameters, that might be useful for monitoring the postvaccination response of patients on anti-CD20 therapy in various age categories.

## 2 Materials and methods

### 2.1 Participants and setting

Patients were enrolled at the Multiple Sclerosis Centre and Myasthenia Gravis Centre of the Department of Neurology at the General University Hospital in Prague. We included 83 MS, 19 NMOSD (all aquaporin-4 antibodies seropositive, AQP4^pos^NMOSD), and 7 MG patients (all acetylcholine receptor antibodies seropositive, AChR^pos^MG) on anti-CD20 treatment and 41 controls (healthcare workers) without any immunosuppressive treatments, who underwent blood sampling for the assessment of SARS-CoV-2-IgG and IFN-γ release following spike protein stimulation. We included individuals who underwent blood sampling at least 4-8 and 16-20 weeks after the second dose of the Pfizer-BioNTech mRNA (BNT162b2) vaccine (baseline time point – M0 and M3 timepoint, respectively). The interval between the first and second dose of the mRNA vaccine against SARS-CoV-2/COVID-19 ranged from 21 to 58 days. The dosing regimen for RTX consisted of two infusions of 1000 mg of RTX two weeks apart, followed by a single infusion of 1000 mg of RTX every 6-7 months in NMOSD and MS patients. The interval of retreatment was longer in MG patients. The OCR treatment was two 300 mg infusions, two weeks apart, followed by a single infusion of 600 mg of OCR every 6-7 months. In general, routine blood and flow cytometry measurements of particular subsets of leukocytes were included in the analysis. All adverse reactions and COVID-19 histories were recorded *via* the national registry ReMuS from March 1, 2020, to December 31, 2021. COVID-19 infection following complete vaccination was analysed up to December 31, 2021. This study was approved by the Ethics Committee of the General University Hospital in Prague (102/21 S-IV and 43/21 S/IV). All participants provided signed written informed consent. This study was conducted according to the Helsinki Declaration.

### 2.2 SARS-CoV-2 IgG targeting spike protein

The serum concentration of the specific immunoglobulin G (IgG) against the trimeric spike glycoprotein of the SARS-CoV-2 virus was measured by a commercially available chemiluminescence immunoassay (CLIA) (LIAISON^®^ SARS-CoV-2 TrimericS IgG, DiaSorin, Italy) following the manufacturer’s instructions. This assay determines the concentration of the SARS-CoV-2 specific IgG in the range between 1.85 and 800 AU/mL and has a high sensitivity and specificity. According to the manufacturer’s data, it also correlates with the presence of neutralizing antibodies. Sera were separated by centrifuging coagulated blood, which was then directly analysed. A manufacturer’s cut-off value of 13 AU/mL was used to differentiate between seronegative or seropositive individuals.

### 2.3 SARS-CoV-2 interferon-gamma release assay

The T cell immune response to SARS-CoV-2 was assessed by detecting IFN-γ using the commercially available Euroimmun SARS-CoV-2 IGRA from Luebeck, Germany. Briefly, test specimens were added to 3 different tubes, 2 of them were precoated with the S1 domain of the spike protein SARS-CoV-2 antigens or with mitogen, and the plain ones (Nil tube) were provided as controls by the manufacturer. The tested whole blood was incubated for 24 h at 37°C, then the blood was centrifuged (12,000 × *g*, 10 min) and the plasma was collected for the detection of IFN-γ using an enzyme-linked immunosorbent assay with an ELISA-based platform (Quan-T-Cell ELISA, Luebeck, Germany). A 100 mIU/mL cut-off value was used to discriminate positive from negative cell-mediated immune responses to the SARS-CoV-2 spike protein.

### 2.4 Lymphocyte subpopulations

In addition to a complete blood count, 50µl of whole blood was taken using EDTA Vacutainer tubes, stained by monoclonal antibodies (mAb) (Beckman Coulter): CYTO-STAT CD45 (FITC), CD56 (RD1), CD19 (ECD), CD3 (PE-Cy5) + mAb CD16 (PE), CD4 (PB), CD8 (PE-Cy7), CD45RA (PE), CD127 (FITC), CD25 (ECD), TCR g/d (PE-Cy5.5), HLA-DR (APC), CD8 (APC-Alexa Fl. 700), CD3 (APC-Alexa Fl. 750), CD4 (PB), IgD (FITC), CD16+CD56 (PE), CD19 (ECD), CD5 (PE-Cy5.5), CD27 (PB), CD45 (KO) and fully processed in a Beckman Coulter Tq-Prep using the Immunoprep Reagent System (lyse no-wash process). Afterwards, the samples were immediately analysed in a Beckman Coulter Navios System flow cytometer. Lymphocytes were gated using SSc vs. CD45 dot plots. This analysis was only performed at time point M0.

### 2.5 Statistical analysis

Firstly, we tested the normality of distribution for individual variables with the Shapiro-Wilk test on a level of significance of p ≤ 0.05. The variables did not have a normal distribution; therefore, in our descriptive statistics, we used median and range and the nonparametric Wilcoxon rank-sum test (WRT) with Spearman’s rank correlation. False discovery rate, according to Benjamini and Yekutieli, was used to prevent multiple comparison problems (critical p-value > 0.0337). Linear regression analysis was performed in different subgroups (MS-ocrelizumab, MS-rituximab, NMOSD, MG, and controls) for descriptive variables (age, sex, treatment duration, corticosteroids dosage, disease duration, and vaccination interval), any significant correlation is mentioned. Due to the heterogeneity of the dataset, we decided to use a combination of two multivariate methods: principal component analysis (for a reduction of dimension, the first 3 dimensions contained 99.92% variance) and DBscan cluster analysis. The condition for individual clusters was defined as minimal points in cluster 3 and the number of outliers was below 5% of the data set. Each cluster was tested by WRT for differences in levels of measured parameters in comparison with the rest of the dataset. All calculations were processed using MATLAB R2018b statistic tools (MathWorks).

## 3 Results

### 3.1 Cohort characteristics

We enrolled a total of 150 participants (66% female), with the first blood collection at a median of 5.14 weeks after the second vaccine dose. Local injection site reactions and systemic events (mostly influenza-like symptoms) were generally mild to moderate, and transient. Patients on RTX had significantly lower side effects following the first dose of the vaccine compared to the other study subjects (p<0.001). Sixty-two MS patients were treated with OCR. The RTX-treated subjects included 47 patients (MS n=21, NMOSD n=19, and MG n=7). There was no significant difference in disease duration, RTX/OCR treatment duration, or severity of neurological impairment as measured by EDSS between the NMOSD and MS groups. Patients being treated for myasthenia gravis were non-significantly older (median 65.33 years) in comparison to other groups (medians between 43.83 to 45.67 years), but this was influenced by the relatively small MG group size. The median time between the last anti-CD20 infusion and the first vaccine dose was 153 days (R84-338 days) in ocrelizumab-treated patients. In patients treated with rituximab, the median time between the most recent infusion and vaccination was 144 days (R41-506). Time from the last dose of rituximab until the first vaccine was significantly longer in patients with MG (median 303 days, p<0.001). Thirteen NMOSD patients were treated with a low dose of corticosteroids (less than 10 mg of prednisolone per day) in addition to RTX. Furthermore, all MG patients were treated with a low dose of corticosteroids (less than 10 mg of prednisolone per day) and other immunosuppressive drugs (azathioprine n=2, tacrolimus n=2, mycophenolate mofetil n=2, and cyclosporin n=1). The characteristics of all the patients and controls are summarized in **Table 1**.

**Table 1:** Patients’ and controls’ distribution, overall demographic, and clinical characteristics.

CHARACTERISTICS	MS (n=83)	NMOSD (n=19)	MG (n=7)	Controls (n=41)
Demographic*				
Age, median (range), y	43.33 (21.08-70.83)	45.33 (28.17-73.92)	65.33 (37-83.42)	45.67 (20.58-79.83)
Female, n (%)	55 (66.26)	15(78.95)	2 (28.57)	27(65.85)
Disease duration, median (range), y	15.25 (2-36.25)	10.25 (1.67-41.25)	9.08 (1.92-22.25)	NA
EDSS, median (range), score	3.5 (0–8)	4 (1.5-7.5)	NA	NA
QMGS, median (range), score	NA	NA	4 (0-15)	NA
Individual anti-CD20, n (%)				
Rituximab	21 (25.3)	19 (100)	7 (100)	NA
Ocrelizumab	62 (74.7)	0	0	NA
Concomitant low dose corticosteroids	0	13 (68.42)	7 (100)	NA
Concomitant other immunosuppressive drugs	1 (1.2)	0	7 (100)	NA
Duration of ocrelizumab/rituximab treatment (range), y				
Rituximab	3 (1.25-5.58)	2.92 (0.75-8)	2.25 (0.75-8)	NA
Ocrelizumab	2.83 (0.75-9.58)	NA	NA	NA
COVID-19 prior vaccination n (%)				
Rituximab	4 (4.82)	1(5.26)	2(28.57)	NA
Ocrelizumab	15 (24.19)	NA	NA	NA
No treatment	NA	NA	NA	4 (9.76)
COVID-19 after 2^nd^ dose of vaccine n (%)	11 (13.25)	3 (15.79)	1 (14.29)	3 (7.31)
Time between the last dose of ocrelizumab/rituximab and 1^st^ vaccine, median (range), days	152 (41-338)	140 (105-206)	303 (143-506)	NA
Rituximab	147 (41-229)	140 (105-206)	303 (143-506)	NA
Ocrelizumab	153 (84-338)	NA	NA	NA
Side effects after 1^st^ dose of BNT162b2 vaccine (mild+moderate/severe)	2.41/0%	5.26/0%	0/0%	NA
Side effects after 2^nd^ dose of BNT162b2 vaccine (mild+moderate/severe)	4.82/0%	10.53/0%	0/0%	NA

*There was no statistically significant between-groups difference in basic demographic characteristics except higher male predominance in the MG group (p=0.0047).

EDSS, expanded disability status scale score; MG, myasthenia gravis; MS, multiple sclerosis; NA, not applicable; NMOSD, neuromyelitis optica spectrum disorder; QMGS, quantitative myasthenia gravis score; y, years.

### 3.2 Humoral responses following SARS-CoV-2 mRNA vaccination in anti-CD20 treated patients with MS, NMOSD, MG, and immunocompetent controls

#### 3.2.1 Serological analysis at baseline (M0, between 4 to 8 weeks after 2^nd^ dose of vaccine)

**Figure 1:**
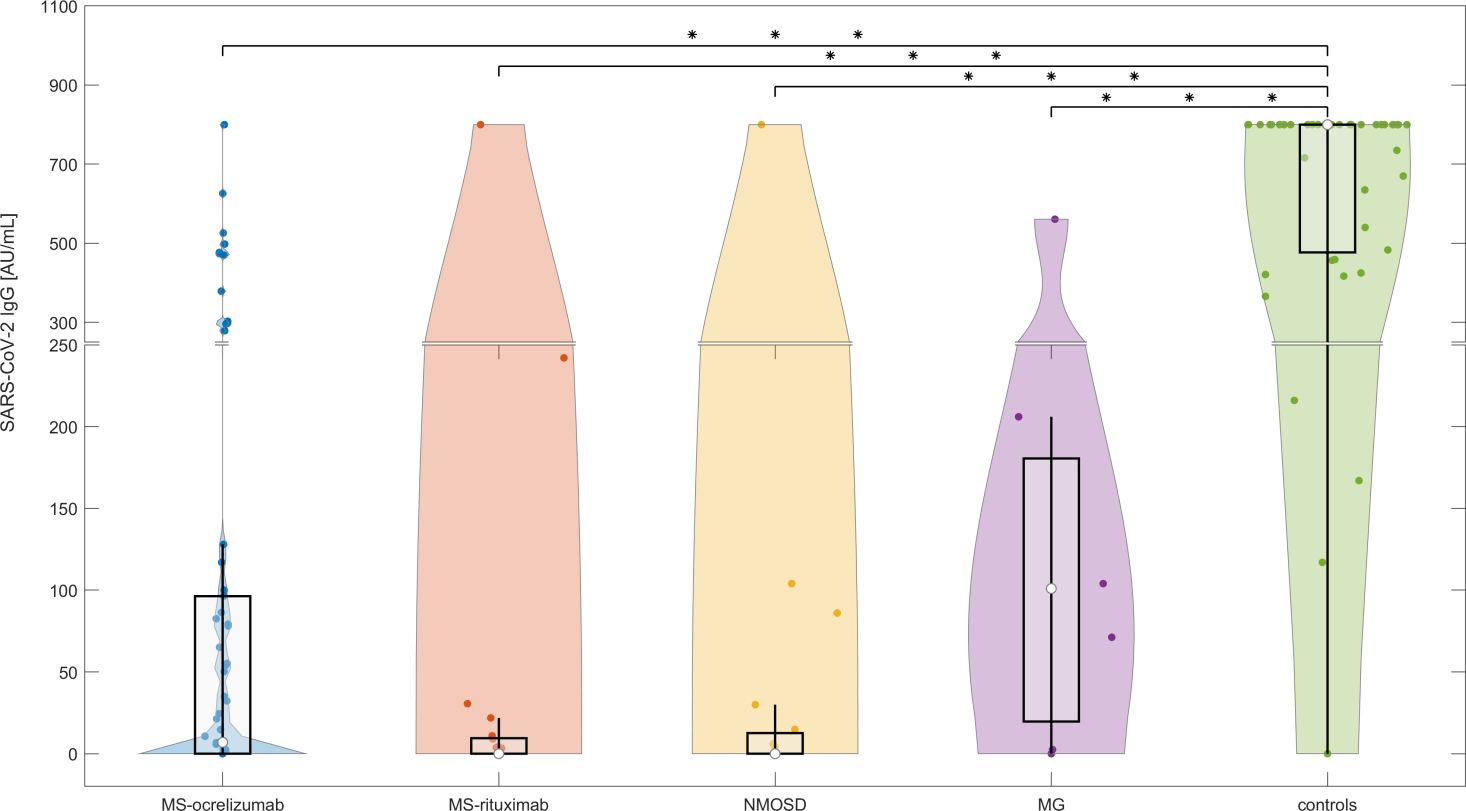
The serological response following SARS-CoV-2 mRNA vaccination in anti-CD20 treated patients with MS, NMOSD, MG, and immunocompetent controls at baseline (M0). The concentrations of post-vaccination SARS-CoV-2 IgG at baseline (4-8 weeks following 2^nd^ dose of vaccine). The boxes show IQR, the median values are represented by white dots, the upper whiskers show Q3 + 1.5 IQR, the lower whiskers show Q1-1.5 IQR, and the colored areas represent probability distributions (kernel density plot). The asterisk is a sign of significance [*** means p-value < 0.001]. MS-ocrelizumab (MS patients treated with ocrelizumab), MS-rituximab (MS patients treated with rituximab), NMOSD = neuromyelitis optica spectrum disorders, MG = myasthenia gravis. Y-axis is broken at a value 250 AU/mL and the axis division in the lower part is 50 AU/mL and in the upper part 200 AU/mL.

The proportion of patients on anti-CD20 treatment who showed a humoral immune response against the SARS-CoV-2 spike protein at least 4 weeks after completion of SARS-CoV-2 vaccination is 34.09% (n=30). In comparison, 97.22% of controls exhibited seropositivity for SARS-CoV-2 IgG in their serum after vaccination. Patients and controls who had experienced COVID-19 before vaccination were excluded from this seroconversion analysis. Anti-CD20 treated patients had significantly lower detectable levels of anti-spike antibodies (median SARS-CoV-2 IgG 2.5 vs. median of controls 800 AU/mL, p<0.0000001). Patients treated with ocrelizumab had a higher seropositivity rate (20/48; 41.67%) compared to those treated with rituximab (10/40; 25%). There was no significant difference in SARS-CoV-2 IgG levels between RTX and OCR-treated patients at this time point (**Figure 1**). The SARS-CoV-2 IgG humoral response was significantly higher in individuals who had completed the vaccination in a period of more than 3 weeks (n=96) in comparison with a group who had completed the vaccination within 3 weeks (n=30) (median SARS-CoV-2 IgG 32.86 vs 0 AU/mL, respectively, p=0.0134). Ocrelizumab and RTX treatment led to a significant risk of seronegativity: OR = 49 CI 95% (6.19 - 387.99), p<0.0000001, and OR 105 CI 95% (12.7 - 868.39), p<0.000000001, respectively.

Comparative univariate analysis of seropositive and seronegative patients did not reveal any statistical significance for the time between the last OCR/RTX administration and the first vaccine. The duration of therapy was significantly longer in non-seroconverted patients (median 36 months) than in seroconverted (median 24 months), with p<0.0001 for RTX and p=0.005 for the OCR-treated group, respectively. We did not find any significant relationship between levels of CD19^+^ B cells and seroconversion. There was no correlation between the proportion of CD19^+^cells or corticosteroids daily used and SARS-CoV-2 IgG antibody levels in different subgroups of patients.

#### 3.2.2 Serological analysis at M3 (between 16 to 20 weeks following 2^nd^ dose of mRNA vaccine)

**Figure 2:**
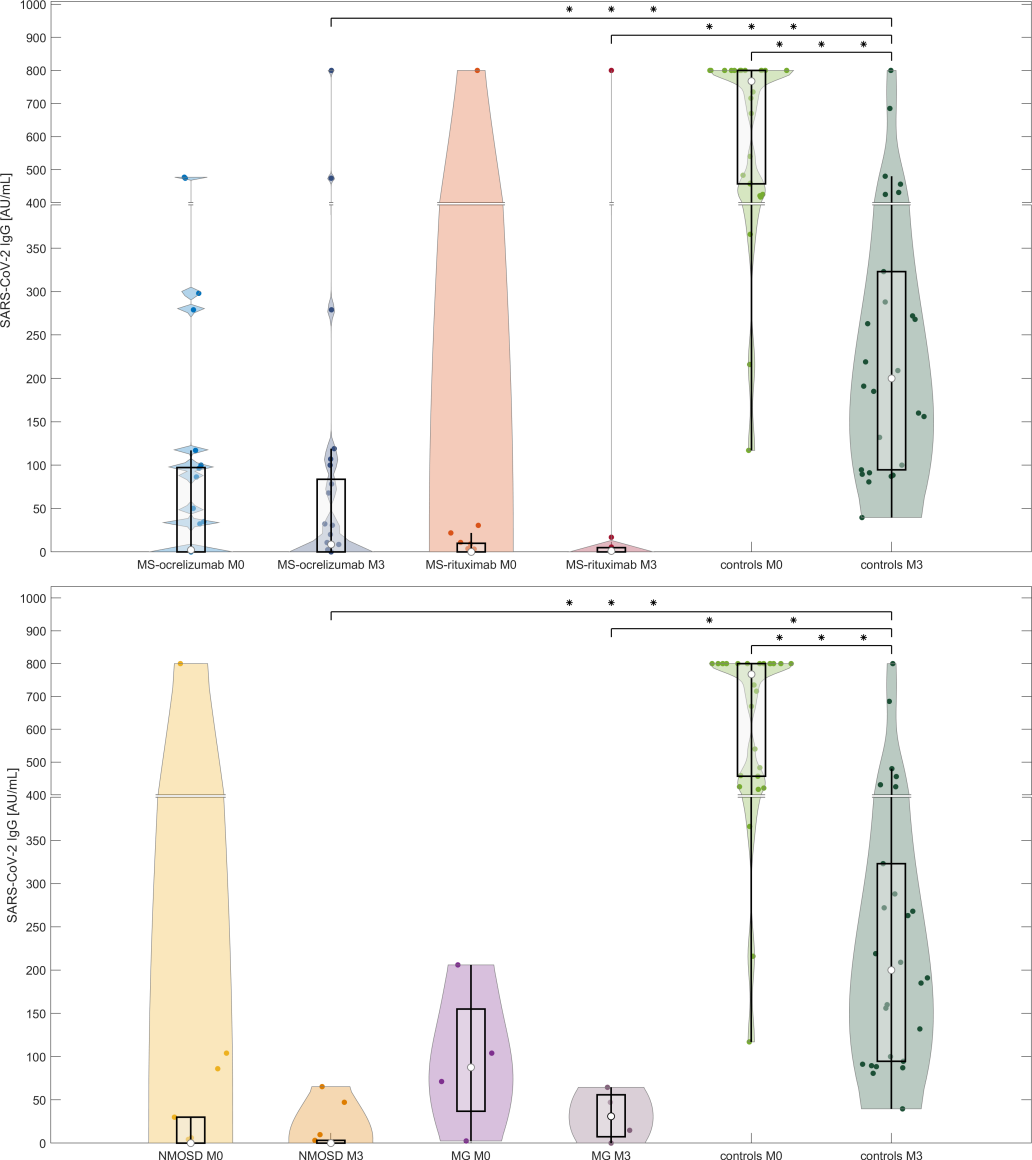
Comparison of antibody responses following SARS-CoV-2 mRNA vaccination in anti-CD20 patients with MS, NMOSD, MG, and immunocompetent controls at two-time points (M0 and M3). The serological response to two doses of BNT162b2 in each treated group and controls at two different time points (4-8 weeks and 16-20 weeks (M3) following 2^nd^ dose of vaccine). The boxes show IQR, the median values are represented by white dots, the upper whiskers show Q3 + 1.5 IQR, the lower whiskers show Q1-1.5 IQR, and the colored areas represent probability distributions (kernel density plot). Asterisk is a sign of significance [*** means p-value < 0.001; ** means p-value < 0.01]. MS-ocrelizumab (MS patients treated by ocrelizumab), MS-rituximab (MS patients treated by rituximab), NMOSD = neuromyelitis optica spectrum disorders, MG = myasthenia gravis. Y-axis is broken at a value 400 AU/mL and the axis division in the lower part is 50 AU/mL and in the upper part 100 AU/mL.

The levels of SARS-CoV-2 IgG decreased significantly in controls over the reference period (median SARS-CoV-2 IgG at baseline (M0) 767.5 AU/mL vs. 200 AU/mL at M3, respectively, p<0.000001). We also observed a decline in the concentrations of antibodies in the NMOSD and MG groups, but this was not significant. On the other hand, the levels of SARS-CoV-2 IgG increased significantly in MS patients treated with OCR (median SARS-CoV-2 IgG at baseline (M0) 2.3 vs. 8.6 AU/mL at M3 respectively, p=0.0014) (**Figure 2**). Levels of IgG SARS-CoV-2 (M3) positively correlated with the time frame of the administration of the 1^st^ and 2^nd^ vaccine doses and levels of SARS-CoV-2 IgG after vaccination (R-square 0.18, Spearman’s rank correlation coefficient 0.32, p=0.0021).

### 3.3 T cell responses after stimulation with spike protein in anti-CD20 treated patients with MS, NMOSD, MG, and controls following SARS-CoV-2 mRNA vaccination

#### 3.3.1 T cell responses to spike protein at baseline (between 4 to 8 weeks after 2^nd^ dose of vaccine)

**Figure 3:**
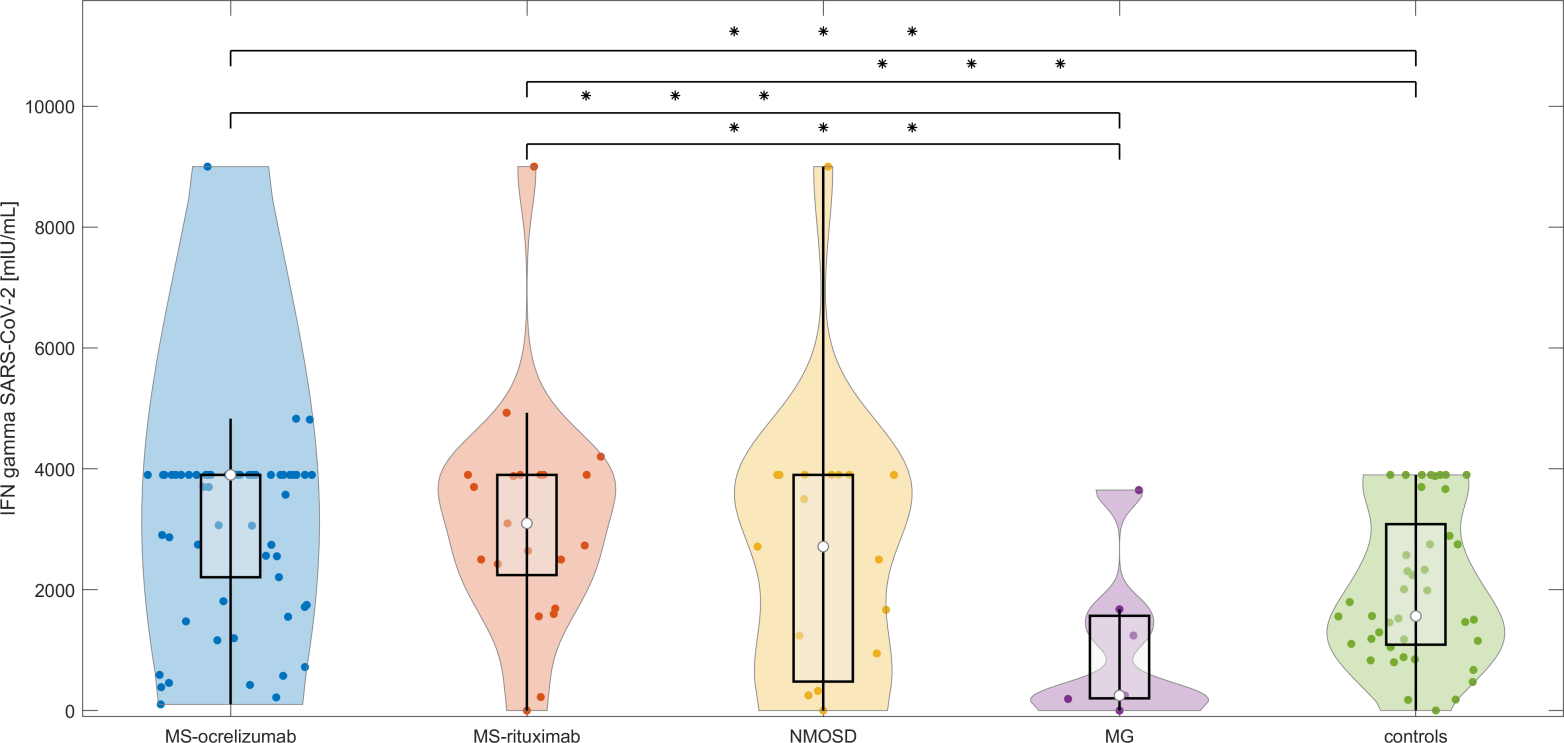
T cell responses after stimulation with spike protein in vaccinated anti-CD20 patients with MS, NMOSD, MG, and immunocompetent controls at baseline. The concentrations of IFN-γ released by peripheral mononuclear cells following spike protein stimulation in each treated group and controls at baseline (4-8 weeks following 2^nd^ dose of vaccine). The boxes show IQR, the median values are represented by white dots, the upper whiskers show Q3 + 1.5 IQR, the lower whiskers show Q1-1.5 IQR, and the colored areas represent probability distributions (kernel density plot). MS-ocrelizumab (MS patients treated with ocrelizumab), MS-rituximab (MS patients treated by rituximab), NMOSD = neuromyelitis optica spectrum disorders, MG = myasthenia gravis. Asterisk is sign of significance [* p-value < 0.05; ** p-value < 0.01; *** means p-value < 0.001].

T cell responses, measured by the production of IFN-γ following spike protein stimulation, were detected in 118/124 (95.16%) participants without a previous history of COVID-19 infection, including 35/40 (87.5%) subjects on RTX, 48/48 (100%) patients on OCR, and 35/36 (97.22%) controls. MS patients had significantly higher detectable levels of released IFN-γ (median SARS-CoV-2 IFN-γ 3572 mIU/mL vs. controls 1545 mIU/mL, p < 0.0014). This was not observed in NMOSD patients. In contrast to MS, the levels of SARS-CoV-2 induced IFN-γ release were lower in MG patients compared with controls (median SARS-CoV-2 IFN-γ 250.5 vs. controls 1545 mIU/mL, p < 0.04) (**Figure 3**). No difference in SARS-CoV-2 IFN-γ levels was detected in individuals who had completed vaccination within 3 weeks in comparison to the group with a vaccination interval of up to 6 weeks. There was no correlation between the proportion of CD19^+^cells or corticosteroids daily used and SARS-CoV-2 IFN-γ levels in different subgroups of patients.

#### 3.3.2 T cell responses to spike protein at M3 (between 16 to 20 weeks after 2^nd^ dose of mRNA vaccine)

**Figure 4:**
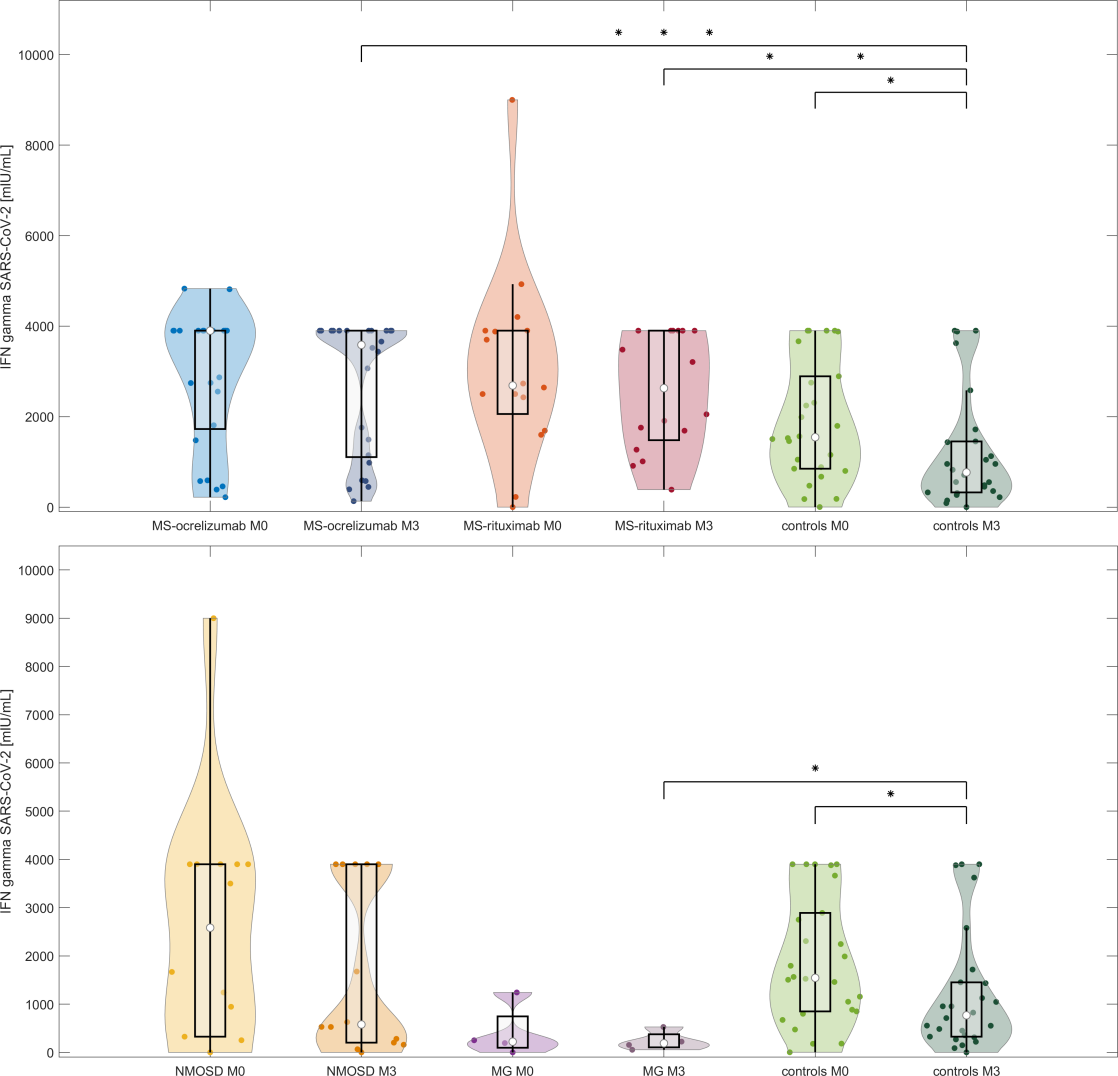
IFN-γ release after stimulation with spike protein in vaccinated anti-CD20 treated patients with MS, NMOSD, MG, and controls at two-time points (M0 and M3). The comparison of concentrations of IFN-γ released by peripheral mononuclear cells following spike protein stimulation in each treated group and controls at baseline and M3 (16-20 weeks following 2^nd^ dose of vaccine). The boxes show IQR, the median values are represented by white dots, the upper whiskers show Q3 + 1.5 IQR, the lower whiskers show Q1-1.5 IQR, the colored areas represent probability distributions (kernel density plot), MS-ocrelizumab (MS patients treated by ocrelizumab), MS-rituximab (MS patients treated by rituximab), NMOSD = neuromyelitis optica spectrum disorders, MG = myasthenia gravis. Asterisk is a sign of significance [* p-value < 0.05; *** means p-value < 0.001].

T cell responses measured by the release of IFN-γ after spike protein stimulation decreased in all patient groups and controls without a history of COVID-19 infection over the reference period (**Figure 4**). When we evaluated the responses in the MS group, we observed a decrease from a median SARS-CoV-2 IFN-γ 3700 to 3480.7 mIU/mL, p=0.4. Patients treated with OCR decreased from a median 3900 mIU/mL to a median of 3586 mIU/mL (p = 0.46). This was in contrast to RTX-treated MS patients, whose responses were almost stationary (median SARS-CoV-2 IFN-γ at M0 2687.8 mIU/mL vs 2629.4 mIU/mL at M3, p = 0.6). NMOSD patients decreased in this parameter most noticeably (median SARS-CoV-2 IFN-γ 2584.1 at baseline to 578.6 mIU/mL at M3, p = 0.43). In healthy controls, the median values dropped by approximately half (from 1545 to 768.1 mIU/mL, p = 0.3).

### 3.4 Cluster analysis according to SARS-CoV-2 IFN-γ and SARS-CoV-2 IgG values

#### 3.4.1 Cluster analysis at time point M0

**Figure 5:**
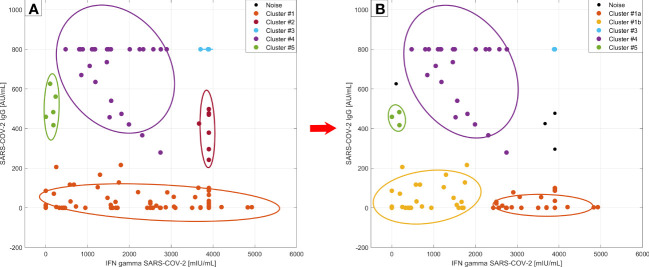
Cluster analysis according to SARS-CoV-2 specific humoral and cellular responses at timepoint M0. DBscan cluster analysis was performed on the whole studied cohort including the subjects with COVID-19 infection prior to vaccination (left side –**A**) and without those subjects (right side –**B**) (ϵ=0.4, minimal points per cluster 3) Legend: • - outliers (3 of them are not plotted, they had IFN gamma values over 4000 mlU/mL); left side –A: • - cluster 1: dominantly anti-CD20 treated patients, • - cluster 2: vaccinated subjects with a COVID-19 infection history and with Treg upregulation,• - cluster 3: immunocompetent controls with maximal humoral/cellular response, • - cluster 4: rest of controls, • - cluster 5: patients without adverse effects from the vaccination; right side - B: • - cluster 1a: new cluster: with more adverse effect reactions after vaccination, • - cluster 1b: higher SARS-CoV2- IgG production than 1a; • cluster 3, • - cluster 4, and • - cluster 5 – remain the same, do not differ from part A.

We were interested in whether there are different phenotypes/profiles of SARS-CoV-2 immunity in the context of vaccination and the other following parameters. We applied DBscan cluster analysis to the first two components of the principal component analysis (PCA) of these variables: SARS-CoV-2 spike protein induced IFN-γ release, SARS-CoV-2 specific IgG, the proportion of lymphocyte subpopulation including regulatory T cells. Cluster analysis distinguished 5 different regions and 3 outliers (**Figure 5A**). The first cluster consisted of 86 anti-CD20 treated patients and 4 immunocompetent subjects with low SARS-CoV-2 IgG (below 220 AU/mL), and a broad range of SARS-CoV-2 IFN-γ levels (0 – 4927.25 mIU/mL). The second cluster included 6 patients and 1 control, who had a high level of SARS-CoV-2 IFN-γ (higher than 3500 mlU/mL) and medium values of SARS-CoV-2 IgG (242-500 AU/mL). These subjects mostly had been diagnosed with a COVID-19 infection prior to vaccination and some of them had had severe disease courses with pneumonia. Furthermore, regulatory T cells (Tregs) had been upregulated in this subgroup (median 10.01% vs median 8.58%, p=0.023). The third cluster consisted of 8 controls and 2 MS patients, who developed a high immune response in both parameters (higher than 3700 mlU/mL of SARS-CoV-2 IFN-γ and 800 AU/mL SARS-CoV-2 IgG). They had longer intervals between both doses of the vaccine (median 42 days compared to 27 days, p = 0.0021). The fourth cluster (n=27) included 25 controls and 2 MS patients sorted according to higher levels of SARS-CoV-2 IgG (more than 270 AU/mL) and SARS-CoV-2 IFN-γ below 2895 mlU/mL. They also had longer intervals between both doses of the vaccine (median 42 vs. 24 days, p < 0.0001). Lastly, the fifth cluster consisted of 3 controls,1 MS patient, and 1 myasthenia gravis patient (0-250 mlU/mL of SARS-CoV-2 IFN-γ and 400-650 AU/mL SARS-CoV-2 IgG). Those patients had no adverse effects from the vaccination.

If we exclude subjects with a history of COVID-19 infection from this analysis we can observe the division of the first cluster into two new clusters with 50 members (41 MS, 8 NMOSD, and 1 control, **Figure 5B**, cluster 1a) and 28 members (12 MS, 8 NMOSD, 5 MG, and 3 controls) (**Figure 5B**, cluster 1b). In the first new cluster, individuals were characterized by SARS-CoV-2 IFN-γ values higher than 2000 mlU/mL. These patients had more severe, adverse effects after vaccination (p = 0.0089). The new cluster 1b had a higher value of SARS-CoV-2 specific IgG than the first cluster (median SARS-CoV-2 IgG 14.75 vs. 0 AU/mL, p=0.0004) and SARS-CoV-2 IFN-γ below 2000 mIU/mL. The previously described second cluster (**Figure 5A**) with high SARS-CoV-2 IFN-γ production and upregulation of Treg disappeared in this analysis, because the majority of those subjects had suffered from COVID-19 previously. In summary, the first group (cluster 1a) of subjects had low levels of SARS-CoV-2 IgG and production of IFN-γ following spike protein stimulation that was higher than 2000 mlU/mL as well as more adverse events. The second group (cluster 1b) was characterized by slightly higher levels of antibodies and production of IFN-γ below 2000 mlU/mL. We have not found any other relationships between lymphocyte subpopulations and markers of humoral or cellular responses.

#### 3.4.2 Cluster analysis of SARS-CoV-2 IFN-γ production

**Figure 6:**
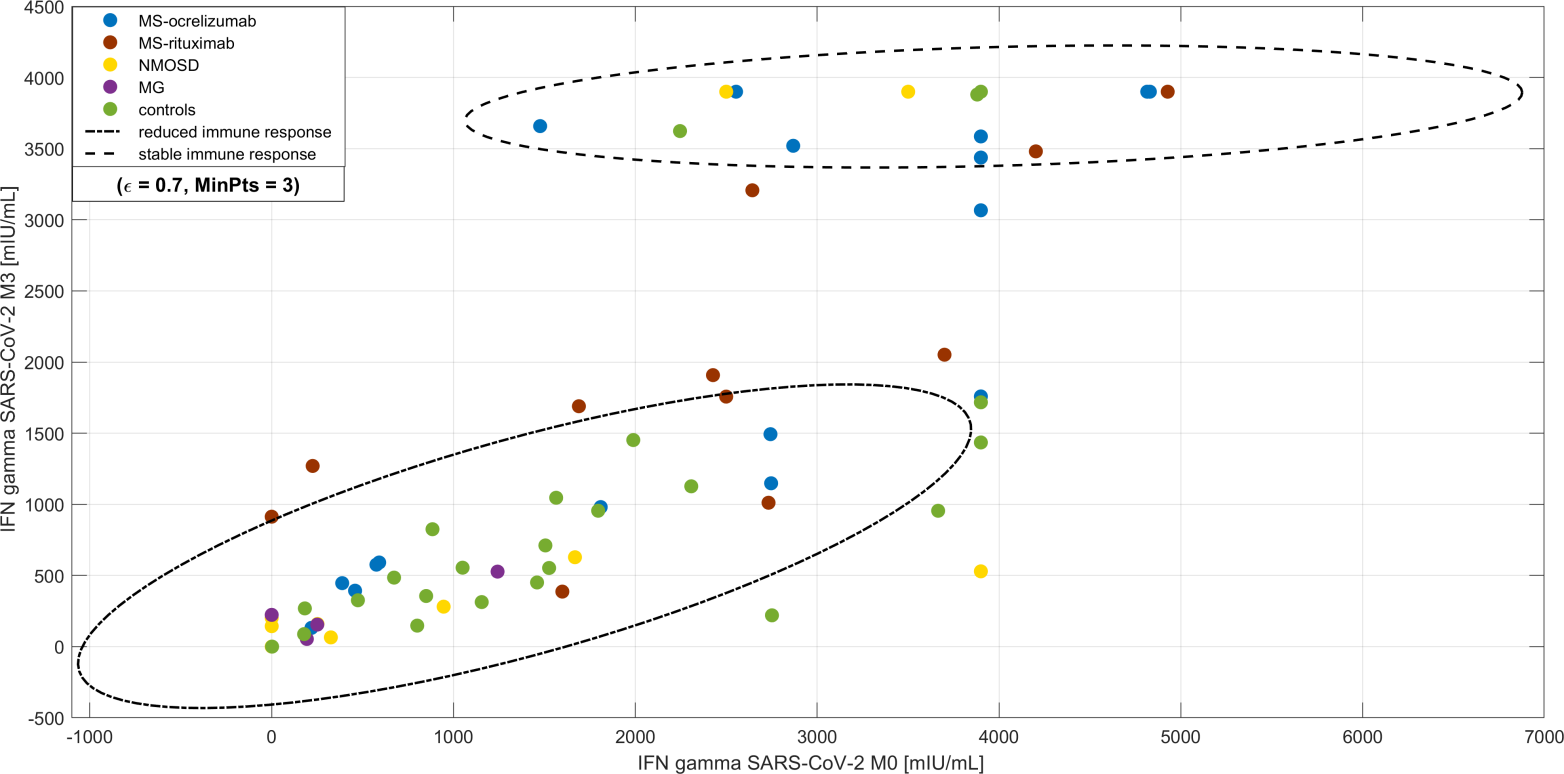
Cluster analysis of longevity of SARS-CoV-2 IFN-γ production upon spike protein stimulation of peripheral blood cells. DBscan cluster analysis was performed on the whole studied cohort including the subjects with COVID-19 infection prior to vaccination (ϵ=0.7, minimal points per cluster 3). There are two clusters, the first is represented by subjects with decreased levels of IFN-γ production upon spike protein stimulation of peripheral blood cells (highlighted by -.- line) compared with a cohort of almost stationary levels (highlighted by - - line). Most of this cohort consisted of patients with multiple sclerosis (MS) and the proportion of adverse effects after vaccination was higher. NMOSD = neuromyelitis optica spectrum disorders, MG = myasthenia gravis.

When we compared the cluster analysis values of SARS-CoV-2 IFN-γ at two different time points following vaccination (M0 and M3) there were 2 recognized clusters. The first cluster consisted of 50 subjects, who had decreased at directly proportional levels after vaccination. The second cluster included 30 individuals (22 MS patients, 4 NMOSD, and only 4 controls). These patients had almost stationary levels of IFN-γ production upon spike protein stimulation of peripheral blood cells (**Figure 6**). Furthermore, these patients often had more adverse events after vaccination (p < 0.001). There was no difference in the time frame between the 2^nd^ dose and blood sampling in both groups.

### 3.5 Clinical effect of immunization

We retrospectively analysed the presence of COVID-19 infections in our study cohort before December 31, 2021, when the Delta variant of SARS-CoV-2 dominated our region. We found that 11 MS (13.25%, two of them were reinfected), 3 NMOSD (15.79%), 1 MG (14.29%), and 3 controls (7.31%) suffered from symptomatic COVID-19 confirmed by PCR tests following vaccination by two mRNA vaccine doses. Monoclonal antibodies to the SARS-CoV-2 virus were administered to neurological patients based on knowledge of impaired post-vaccination response due to anti-CD20 treatment and the risk of severe COVID-19 due to these treatments. So far, we are not able to evaluate the protection of vaccination against the severe course of SARS-CoV-2 infection, but none of our patients were admitted to a hospital due to COVID-19 when the primary and secondary preventions against the severe course of the infection were used.

## 4 Discussion

Vaccination against the SARS-CoV-2 virus and monitoring of immune response are important parts of the management of patients with autoimmune neurological disorders. We studied the postvaccination response in patients with MS, AQP4^pos^NMOSD, and AChR^pos^MG undergoing anti-CD20 treatment.

We found that 34.09% of patients without a medical history of COVID-19 infection prior to vaccination developed positive SARS-CoV-2 specific IgG. However, the levels of these antibodies were very low in most of our patients. The proportion of seroconverted patients on anti-CD20 therapy can differ between published works. This fact can also be influenced by the method of antibody assessment, the different time spans between the infusion administration and vaccination, and the type of vaccine administered. We did not confirm a link between the time period from the last infusion until the vaccination, and humoral responses. This can be due to the relatively short time frame between the last infusion administration and vaccination in our MS and NMOSD cohorts (approximately 5 months) in contrast to published works (4, 19). Sormani et al. have shown the necessary time span between the last infusion and antibody response to the vaccine is 143 days (95%CI=84-258), but the cohort consisted of patients who had also been vaccinated by the other mRNA vaccine that has a supposedly higher impact on humoral response. Disanto et al. have also shown a progressive increase in SARS-CoV-2 IgG over time since the last anti-CD20 infusion, with a noticeable boundary of more than six months (19). It is consistent with the idea that the immunological effect of anti-CD20 treatment may last longer than 6 months. A very low humoral response was detected regardless of diagnosis or age in our patients undergoing anti-CD20 treatment. Duration of the anti-CD20 treatment for fewer than 2 years was positively associated with a higher proportion of SARS-CoV-2-IgG seropositive patients. Whether there is a difference based on the use of RTX or OCR on the time frame for CD19^+^ cell repopulation in the context of vaccination response has not been satisfactorily elucidated yet, but it seems that there might be some difference. In line with the previous work, some patients exhibited antibody responses to the spike protein in the absence of detectable circulating B cells in our cohorts. This fact perhaps points to the early repopulation within the lymphoid tissue of B cells that are capable of contributing to serological responses (12). This could also explain the better humoral response in patients with a longer period between the 1^st^ and 2^nd^ vaccine doses. In addition, we also observed a delayed humoral response in MS patients treated with ocrelizumab, but not in RTX-treated patients. Both drugs, OCR and RTX, can also target CD20^pos^T cells, which are elevated in peripheral blood in different autoimmune disorders (20–22). Whether there is a distinct potential of different drugs to influence this T cell subpopulation should be addressed in future studies.

In many viral infections, CD4^+^ and CD8^+^ T cells are key cellular subsets for the control and clearance of an acute infection. We have shown preserved or upregulated cellular responses to spike protein *ex vivo* in NMOSD and MS patients upon vaccination. This was not observed in MG patients, who were treated with other immunosuppressive drugs alongside RTX. The different arms of adaptive immunity can compensate for each other in protective immunity in some conditions (15). The compensation of decreased humoral response by the other arms of adaptive immunity could be an explanation for the increasing production of IFN-γ in ocrelizumab-treated patients suffering from MS. This compensation was not observed in patients with NMOSD or MG. This fact can be explained by concomitant medication in MG patients. Based on previous studies focusing on vaccine immunogenicity in patients treated with corticosteroids, we can presume that their effect depends on the daily dose. A dose of corticosteroids higher than 10mg/day can affect the response (23). The risk of decreased vaccine efficacy, as measured by humoral or cellular response, increases in patients on combined immunosuppressive therapy in different autoimmune disorders (24, 25).

The group of MG patients was very small, therefore all results for such a group must be interpreted very carefully. NMOSD and MG patients were non-significantly older but immunosenescence must be taken with caution as well. We presume that the effect of age on the immunogenicity of vaccination was not seen due to the strong impact of anti-C20 therapy on the immune response. We also cannot exclude the role of different immune mechanisms that are related to the pathology of each disease. There is evidence that RTX has better efficacy in controlling disease activity in MG patients with autoantibodies against muscle-specific kinase compared to those who have AChR-IgG (26).

It seems that the longevity of specific cellular responses to spike proteins is different and can last longer in subgroups of MS or NMOSD patients in comparison to healthy controls. Whether cellular response can protect patients on anti-CD20 therapies against the severe course of COVID-19 in our observed cohorts remains unanswered, because the administration of monoclonal antibodies against SARS-CoV-2 in the case of SARS-CoV-2 infection occurred in the majority of patients. Higher values of SARS-CoV-2 IFN-γ were associated with a higher prevalence of adverse effects following vaccination. Using cluster analysis, we have shown an increased proportion of regulatory T cells in the subgroup of patients who had suffered from COVID-19 prior to vaccination.

We did not observe any severe adverse events from vaccination in the MS, NMOSD, and MG patients in our studied cohort. The presence of mild or moderate adverse events was higher in patients with higher SARS-CoV-2 IFN-γ. Our finding is in line with the previous work of Stastna et al. that risks from COVID-19 infection are more pronounced than risks of vaccination in relation to disease exacerbation (27).

This study has limitations related to the retrospective study design. We did not perform serological analysis prior to vaccination, and we can not exclude a previous asymptomatic SARS-CoV-2 infection in a minority of the studied groups. We did not assess virus-neutralizing antibodies, but we assumed a very good correlation with antibodies to spike protein. Unfortunately, we did not characterize the IFN-γ-producing peripheral T cell in detail and did not include the total IgG or IgM levels in the analysis. The boosting of humoral immunity in patients with MS, NMOSD, and MG treated with anti-CD20 therapy is questionable and certainly needs further observation at different time points. A comparison of patients who experienced COVID-19 to a group of patients who have had a 3^rd^ dose of the vaccine will be very important. Furthermore, more attention should be paid to patients with reinfections despite vaccinations and with other concomitant immunosuppressive therapies. The prolongation of the interval between pulses of anti-CD20 therapy and when the vaccine is administered should be considered in clinically stable patients. Otherwise, the combination of primary and secondary prevention tools should be useful in preventing severe COVID-19 infection in anti-CD20 treated patients. Our data from this pilot study should be confirmed in a larger cohort of MS, MG, and NMOSD patients undergoing distinct therapies in a future study.

## 5 Conclusion

Our data in combination with those already published, can be instrumental in better defining the most appropriate SARS-CoV-2 vaccine strategy in MS, NMOSD, and MG patients treated with anti-CD20 therapy. A longer interval between the 1^st^ and 2^nd^ dose of the vaccine seems to boost seroconversion. However, some humoral responses might still be visible within the first 2 years of anti-CD20 treatment independently of infusion or vaccination interval. There is a higher seroconversion rate in patients treated with ocrelizumab, when compared with the rituximab-treated group. Furthermore, concomitant immunosuppressive treatment alongside anti-CD20 agents influences cellular response.

Assessing IFN-γ production following stimulation of peripheral blood cells by SARS-CoV-2 spike protein is likely the most useful marker of vaccine response in CD20-treated patients. It is worth mentioning that higher production of IFN gamma was associated with a higher rate of adverse vaccine effects in patient cohorts.

## Data availability statement

The raw data supporting the conclusions of this article will be made available on reasonable request by the authors, without undue reservation.

## Ethics statement

The studies involving human participants were reviewed and approved by the Ethics Committee of the General University Hospital in Prague (102/21 S-IV and 43/21 S/IV). The patients/participants provided their written informed consent to participate in this study.

## Author contributions

PN and MT: study concept and design, interpretation of data, and drafting of the manuscript. DS, IM, IR, KD, and MT: acquisition of data. PN, HP, VM, GS, and HK: samples analysis, biobanking, and interpretation of data. AT: statistical analysis. DH, JH, and HP: critical revision of the manuscript for important intellectual content and study supervision. All authors contributed to the article and approved the submitted version.
